# Insulin-like growth factors and aging: lessons from Laron syndrome

**DOI:** 10.3389/fendo.2023.1291812

**Published:** 2023-10-24

**Authors:** Haim Werner, Zvi Laron

**Affiliations:** ^1^ Department of Human Molecular Genetics and Biochemistry, School of Medicine, Tel Aviv University, Tel Aviv, Israel; ^2^ Endocrinology and Diabetes Research Unit, Schneider Children’s Medical Center, Petah Tikva, Israel

**Keywords:** growth hormone (GH), insulin-like growth factor-1 (IGF1), IGF1 receptor, Laron syndrome, aging

## Abstract

The growth hormone (GH)-insulin-like growth factor-1 (IGF1) signaling pathway emerged in recent years as a key determinant of aging and longevity. Disruption of this network in different animal species, including flies, nematodes and mouse, was consistently associated with an extended lifespan. Epidemiological analyses have shown that patients with Laron syndrome (LS), the best-characterized disease under the umbrella of the congenital IGF1 deficiencies, seem to be protected from cancer. While aging and cancer, as a rule, are considered diametrically opposite processes, modern lines of evidence reinforce the notion that aging and cancer might, as a matter of fact, be regarded as divergent manifestations of identical biochemical and cellular underlying processes. While the effect of individual mutations on lifespan and health span is very difficult to assess, genome-wide screenings identified a number of differentially represented aging- and longevity-associated genes in patients with LS. The present review summarizes recent data that emerged from comprehensive analyses of LS patients and portrays a number of previously unrecognized targets for GH-IGF1 action. Our article sheds light on complex aging and longevity processes, with a particular emphasis on the role of the GH-IGF1 network in these mechanisms.

## Introduction to the GH-IGF1 system

Pituitary-derived growth hormone (GH) along with insulin-like growth factor-1 (IGF1) constitute an endocrine axis with critical roles in growth and development (1–3). The original hypothesis of Salmon and Daughaday, formulated in the late 1950s, claimed that the vast majority of the biological actions of GH are mediated by an hepatic peptide at first termed *somatomedin* and, subsequently, IGF1 (4). IGF1 is evolutionarily and structurally related to insulin. Prenatal IGF1 expression is GH-independent and becomes GH-dependent around the time of birth. After delivery, liver IGF1 production continues to be dependent on hypophysial GH secretion throughout all stages of life (5).

Aging is linked to various endocrine deficits. In the specific context of the somatotrophic axis, GH and IGF1 biosynthesis progressively decrease as we age due to reduced activity of the hypothalamic GH releasing hormone (GHRH)-GH neuroendocrine system (6). Thus, while maximal GH and IGF1 levels are reached at mid-puberty, concentrations around the eight decade of life become drastically reduced (7). Indeed, both the amplitude of the GH secretory pulses as well as the basal levels between pulses are largely decreased (8). Reduction of endocrine GH levels is closely followed by a parallel decline in circulating IGF1.

Evidence has accumulated in recent years demonstrating that disturbance of the GH-IGF1 network correlates with prolonged lifespan in a number of animal species, including flies (*D. melanogaster*), nematodes (*C. elegans*) and mouse (*M. musculus*) (9–11). Male mice harboring a disrupted GH receptor (*GHR*) gene (‘*Laron*’ mice) survive 55% longer than wild-type animals whereas female *Laron* mice have a 38% longer lifespan (12). The cellular and biochemical mechanisms that are responsible for the association between abrogation of the GH-IGF1 axis and prolonged lifespan are complex. Briefly, these mechanisms are functionally linked to the physiological role played by these hormones in nutrient sensing (13). Of relevance, whereas the effect of individual mutations on lifespan and health span in humans is usually difficult to assess, genomic analyses identified several differentially-represented aging-associated genes in Laron syndrome (LS) patients (14–16).

The present review article summarizes recent data concerning the linkage between the GH-IGF1 axis and aging. Our review highlights mechanistic aspects that emerge from genomic, bioinformatic and biochemical analyses of LS patients. These studies identified new, previously unrecognized targets for GH-IGF1 action and shed light on complex aging and longevity processes (17).

## Laron syndrome

Growth retardation in children is linked to multiple factors and conditions. Cases in which no specific genetic, molecular or biochemical defect can be identified are regarded as *idiopathic* (5). *Congenital IGF1 deficiencies* are typically associated with low serum IGF1 but normal to high GH levels (18). IGF1 deficiencies may result from:

(1) GHRH-receptor (*GHRH-R*) defect;
(2) *GH* gene deletion (isolated GH deficiency, IGHD);
(3) GH receptor (*GHR*) gene deficiency (Laron syndrome, LS); and
(4) *IGF1* gene deletion.

Further conditions resulting in congenital IGF1 deficiency are deficiencies of post-GHR signaling (*e.g*., *STAT5* defects), acid-labile subunit (*ALS*) mutations and pregnancy-associated plasma protein A2 (*PPA2*) mutations (19–24). Congenital IGF1 deficiencies provide an exceptional chance to address key physiological and pathological aspects of the GH-IGF1 axis. Even though these diseases are very rare, fundamental paradigms were derived from the analyses of these conditions, colloquially termed ‘*experiments of nature*’ (3, 5, 25, 26).

Laron syndrome is the best described type of IGF1 deficiency under the spectrum of the GH-IGF1 pathologies (27). The main traits of LS children are short stature (-4 to -10 SDS below median), characteristic face, adiposity, elevated serum GH and low IGF1, insensitivity to GH administration (28–30). The identification of a mutated *GHR* gene as the etiological factor underlying LS was first reported in 1989 (31, 32). In subsequent studies, a series of *GHR* gene anomalies were identified (33). These defects included exon deletions and nonsense, frame shift and missense mutations. Regardless of the variations in the *GHR* defects detected, the outcomes in terms of phenotype were highly similar. Finally, broad analyses of the disease over more than fifty years have had a huge impact on our understanding of normal and pathological growth (16, 18, 34).

## Laron syndrome and cancer protection

While a link between *high* IGF1 levels and enhanced cancer risk has been recognized more than twenty-five years ago, a potential protective role of *low* IGF1 dosages has been more difficult to demonstrate (35–38). This last concept has been supported by an epidemiological study conducted on a cohort of congenital IGF1 deficient patients, which revealed a marked reduction in cancer incidence in homozygous patients compared to their heterozygous relatives (39). The analysis included 230 LS patients, 116 patients with IGHD, 79 patients with *GHRH-R* defects, and 113 patients with congenital multiple pituitary hormone deficiency (cMPHD)]. In addition, the study included 752 of their first-degree family members. Among the 230 LS patients, not a single one developed cancer. Among the 116 IGHD patients, only one had a tumor. On the other hand, among first-degree family members (mostly heterozygotes) 30 instances of cancer were reported. Notwithstanding the fact that the total number of patients was modest, differences between patients and relatives were regarded as highly significant in statistical terms. Furthermore, while the total number of LS patients worldwide is unknown, it is estimated that the percentage of LS patients included in this epidemiological survey was 30-40% of the entire worldwide LS population (27, 30, 40).

The epidemiologic proof that patients with LS do not develop cancer is of foremost clinical relevance. This discovery is in agreement with the notion that the somatotrophic axis is of critical importance in the cell’s ‘*decision*’ whether to engage in proliferation or apoptosis (41, 42). Early studies have identified IGF1 as a progression factor that is required for cell cycle transition (43). Moreover, the bioactivities of IGF1 in the chain of events leading from a normal cell to a malignantly transformed one have been, to a large extent, dissected in biochemical terms. The neoplastic traits include: growth factor independence, chromosomal abnormalities, loss of cell-cell contact inhibition, activation of oncogenes, accumulation of mutations, and others (44). The identification of pathways associated with IGF1 action (“*IGF1 signatures*”) will have a great impact on the optimization of therapeutic tools directed against this growth factor system. Furthermore, these analyses will impinge on the ability to predict responsiveness to anti-IGF1R selective drugs (45–48).

## Cancer protection and aging pathways exhibit a major overlap

As alluded to above, over the past decades the GH-IGF1 axis emerged as a critical determinant of aging and longevity. While cancer and aging are generally believed to constitute largely opposite processes, modern lines of evidence support the concept that cancer and aging might be regarded as different outcomes of the same fundamental processes. These processes include, among others, genomic instability, accumulation of cellular damage, *etc* (**Table 1**) (13).

**Table 1:** Common biochemical and cellular processes underlying cancer and aging.

Accumulation of cellular damage	
Genomic instability	
Epigenetic alterations	
Deregulated nutrient sensing	
Mitochondrial dysfunction	
Stem cell exhaustion	
Cellular senescence	
Telomere attrition	

While increased IGF1 levels as well as constitutive activation of the IGF1R are important risk factors in cancer, reduced activities of the GHR, IGF1R, insulin receptor and downstream mediators (*e.g*., AKT, mTOR, FOXO) have been associated with a prolonged lifespan (49). Paradoxically, classical studies have shown an association between GH/IGF1 deficiency and a number of age-related features (50, 51). Some of these traits include thinning of the skin, excess adiposity, reduced muscle mass, reduced physical performance, *etc* (**Table 2**). The fact that features associated with GH deficiency, as detailed above, constitute manifestations of an aging archetype that is, intuitively, opposed to that classically correlated with enhanced longevity suggest the existence of complex underlying signaling networks. Divergent actions of GH and IGF1 might provide, at least in part, a biologically-plausible explanation to the diametrically opposite patterns of aging regulation depicted above. Furthermore, it is clear that it is not always feasible to infer from flies and nematode models into human biology (52). Hence, extreme care should be exerted when performing such extrapolations. The important role of the IGF1 axis in mitochondrial biology and oxidation processes and, particularly, the impact of these processes on senescence is described below.

**Table 2:** Resemblance between GH deficiency and aging.

Thinning of skin (wrinkling)	
Excess of adipose tissue (obesity)	
Decline in β-cell function	
Enhanced insulin resistance (type 2 diabetes)	
Reduced lean body mass (muscle reduction)	
Reduced physical performance	
Reduced mineral density (osteoporosis)	
Elevated serum cholesterol	

## The GH-IGF1 axis and lifespan: studies in humans

The potential impact of the age-associated decrease in GH and IGF1 levels on lifespan and health span has been a matter of debate (53–55). Examination of prospective correlations of serum IGF1 with mortality, vascular disease, dementia, osteoporosis, diabetes and cancer, led to the identification of two general patterns (56). First, younger persons with high IGF1 are, for the most part, protected from disease. In contrast, older individuals with elevated IGF1 are at risk for occurrence of disease or death. Second, the correlation between IGF1 levels and disease risk is U-shaped. Hence, both high and low IGF1 concentrations might be harmful. Cancer, which is generally positively correlated with IGF1 levels, should be regarded as an exception to this U-shaped pattern. As a corollary, IGF1 signaling could be detrimental in older adults. Patients with LS who were not treated with IGF1 constitute a unique prototype for evaluating the impact of genetically low IGF1 on lifespan and health span (30). We can state with a high degree of confidence that lifelong IGF1 deficiency in untreated LS patients does not appear to noticeably prolong their lifespan. On the contrary, if their cardiovascular and metabolic problems are not treated in time, their lifespan might be shortened. In conclusion and despite the absence of definite epidemiological substantiation on longevity in congenital IGF1 deficiencies, the pivotal role of the GH-IGF1 network in the control of lifespan, as described above, has been extensively documented in various animal models.

## Genomic analysis of LS patients identifies TXNIP as a novel IGF1 target gene linked to senescence regulation

**Figure 1:**
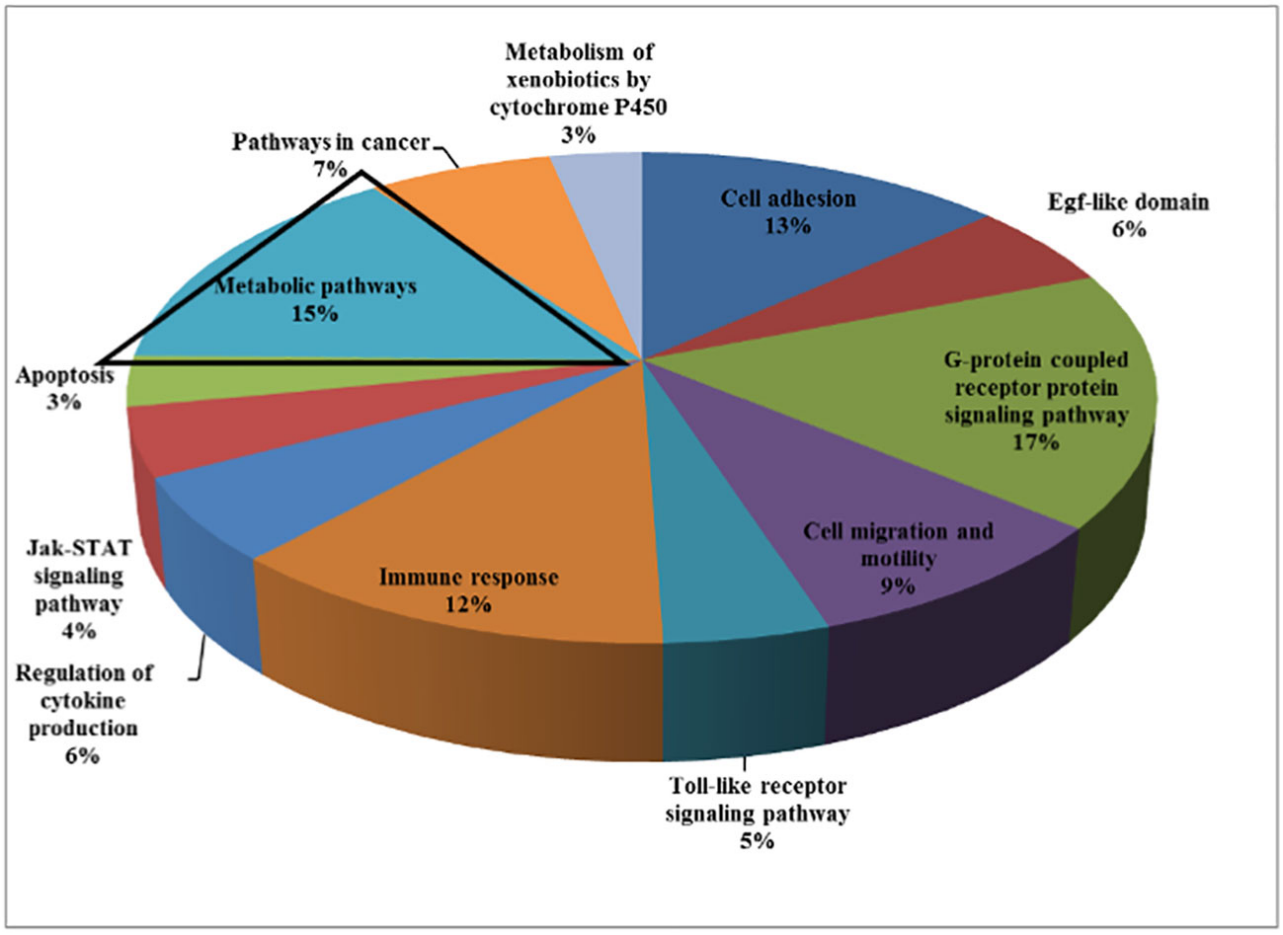
Genome-wide profiling of LS patients. Cluster analysis of differentially expressed genes in LS patients (n =4) compared to healthy controls (n = 3) of the same gender, age, and ethnic origin was conducted. Functional analyses were performed to find co-expressed genes sharing the same pathways. Analyses provide evidence for a number of shared pathways, including cell adhesion, G-protein signaling pathway, cell migration and motility, immune response, Jak-STAT signaling, apoptosis, etc. About 15% of the differentially expressed genes were involved in metabolic pathways. For the most part, genes involved in the control of cell cycle, motility, growth, and differentiation were downregulated in LS-derived lymphoblastoid cell lines compared with controls.

Recently conducted genomic analyses of LS patients reported the identification of differentially expressed signaling pathways and genes in immortalized lymphoblastoid cells. Patients were compared to age-, gender- and ethnicity-matched controls (14, 15). Bioinformatics analyses allowed the clustering of differently expressed genes on the basis of their biological roles (**Figure 1**). Among other biological categories, fifteen percent of the identified genes participated in metabolism. Given the central regulatory role of IGF1 and insulin in the metabolism of cancerous cells, it is logic to assume that adjustments in the expression of metabolic genes may be mechanistically relevant towards the acquisition of a transformed phenotype (16).

The thioredoxin-interacting protein (TXNIP) was identified in genomic analyses as one of the top upregulated genes in LS. TXNIP (57) is an important player in several cellular processes, including metabolism and apoptosis (58–60). For example, TXNIP inhibits glucose uptake, with important consequences in terms of cell metabolism (61). TXNIP stabilizes p16 and p27, two Cdk inhibitors, with ensuing inhibition of cell division (62). Based on these activities, TXNIP is classified as a member of the cell cycle inhibitory enzymes. In agreement with this classification, downregulation of TXNIP is regarded as a prerequisite for cell division. Hence, TXNIP operates as a *bona fide* tumor suppressor (63–65).

**Figure 2:**
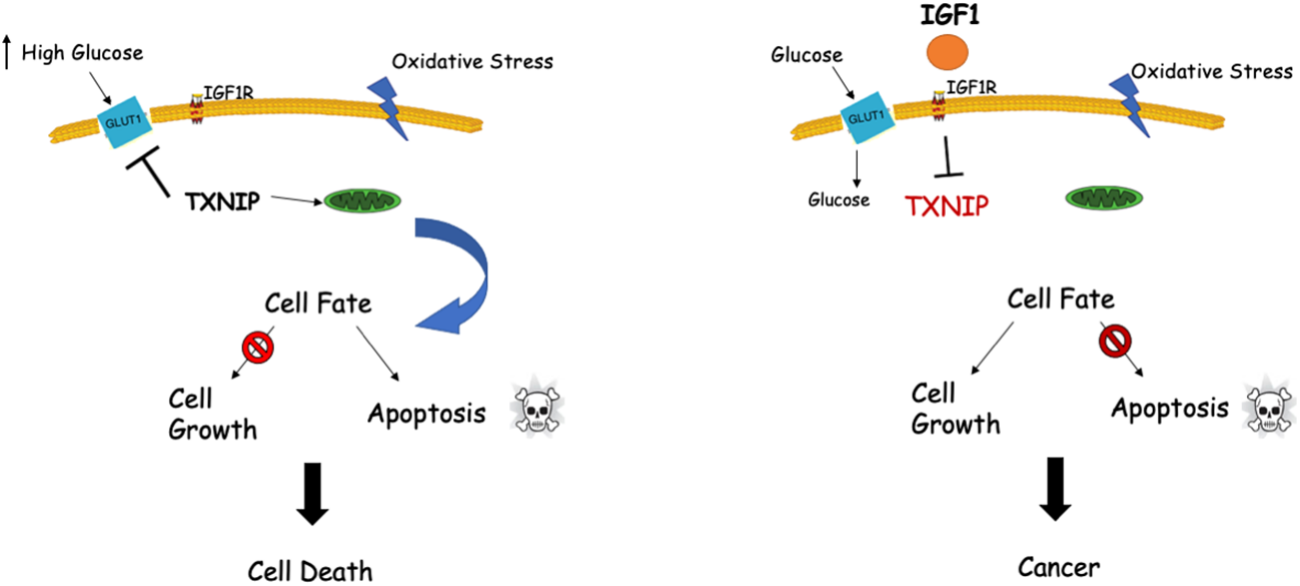
Interplay between IGF1 and TXNIP in regulation of cell survival and homeostasis. TXNIP was shown to be upregulated under normal physiological stress conditions like starvation, oxidative and glucose stresses. Upregulated TXNIP initiates apoptosis by interacting with thioredoxin and translocating to mitochondria (left panel). Cellular stress in the presence of IGF1 (right panel) might lead to marked downregulation of TXNIP levels with ensuing deregulated cell growth, including cancer.

Genomic analyses discovered a functional link between IGF1 and TXNIP (66, 67). Specifically, TXNIP was shown to be expressed at high levels in LS cells. Given that TXNIP has a key role in cellular redox regulation, and in view of the fact that IGF1 controls TXNIP levels under various stress situations (*e.g*., high glucose, oxidative stress), we postulated that the IGF1-TXNIP loop has a crucial role in helping achieve an optimal balance in cellular homeostasis. Our data demonstrated that TXNIP is of vital importance for the cell fate choice, particularly when cells are confronted with different stress signals (**Figure 2**).

**Figure 3:**
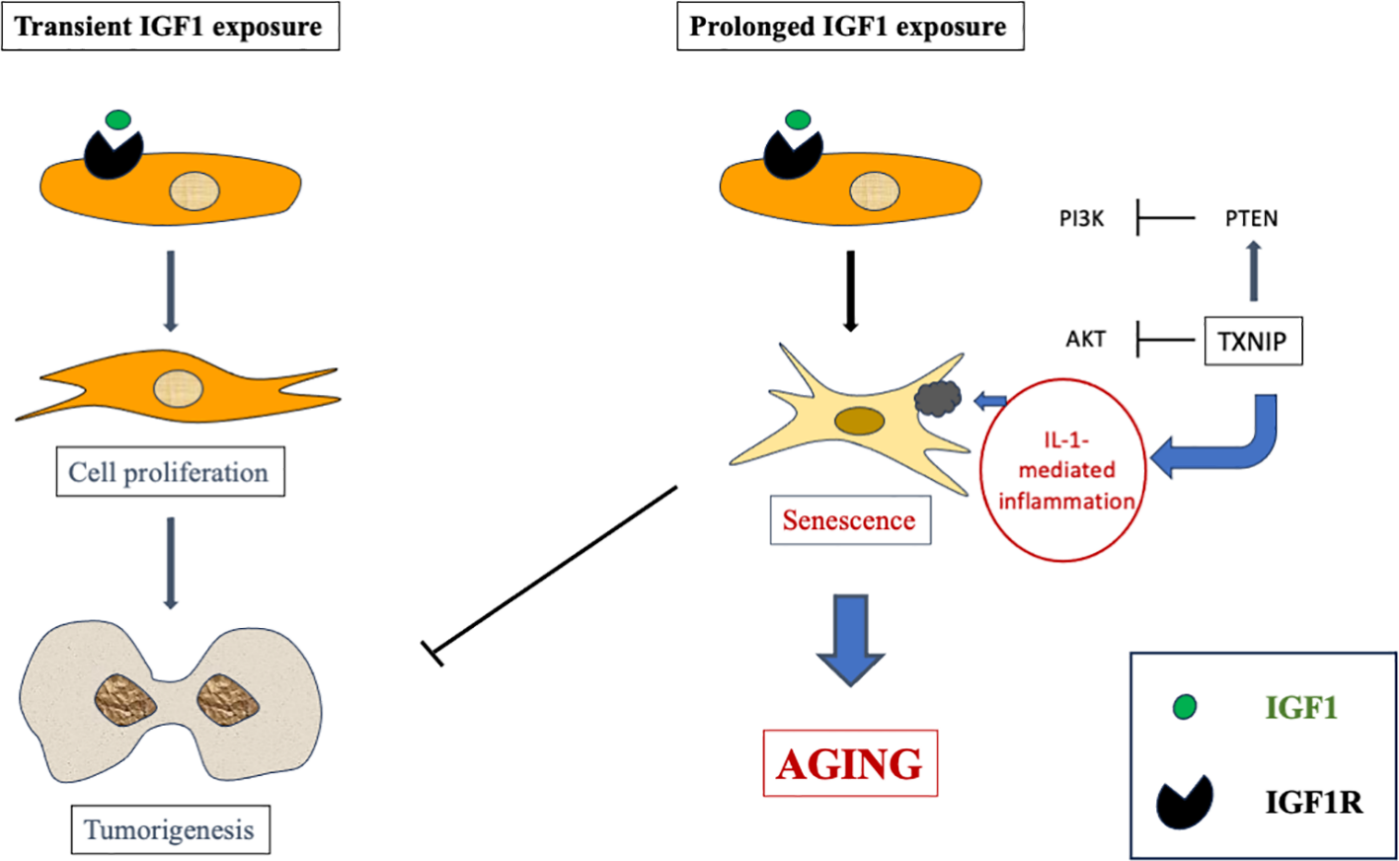
Schematic representation of short- versus long-term IGF1 treatment. Whereas short-term IGF1 stimulation is usually associated with cell proliferation and, potentially, tumorigenesis, prolonged IGF1 stimulation leads to cellular senescence via interaction with mitochondrial protein TXNIP.

The cell state known as *cellular senescence* has been shown in recent years to be implicated in several physiological processes as well as in a number of age-related disorders (68–70). Senescence is usually tied to senescence-associated growth arrest, which is characterized by a senescence-associated secretory phenotype. Our studies have provided evidence that extended IGF1 treatment *in vitro* stimulates the acquisition of a premature senescence phenotype. This phenotype is typified by a unique senescence signature (67). Hence, IGF1 plays a dual role by stimulating mitosis and survival following short-term treatment while inducing premature senescence after long-term exposure (**Figure 3**).

## Laron syndrome is associated with dysregulation of MIR132-3P: impact on aging genes

In addition to the transcriptional analyses depicted in the previous section, genome-wide surveys were conducted to identify microRNAs (miRs) that are differently expressed in LS. We hypothesized that differently represented miRs might account for, at least part of, the phenotypic traits of LS patients. MiRs are endogenous short non-coding RNAs that control the expression of complementary mRNAs (71–73). MiRs pair to specific protein-coding mRNAs, with ensuing post-transcriptional silencing of target genes. miRs are involved in multiple processes. These processes include cell death and proliferation, patterning of the nervous system and hematopoiesis. Finally, a number of miRs that are involved in the modulation of members of the IGF signaling pathway have been identified (74–76).

MiR-132-3p affects a number of biological functions (*e.g*., inflammation, angiogenesis, neuronal differentiation, *etc*) and therefore is considered a key miR (77). Our analyses showed that miR-132-3p is highly expressed in LS. Given that LS is associated with low IGF1 levels, we postulated that miR-132-3p is negatively regulated by IGF1. Bioinformatics analyses helped identify a series of genes whose expression is modulated by miR-132-3p. Lastly, the mechanistic aspects of the IGF1-miR-132-3p regulatory loop are yet to be elucidated.

Using genome-wide analyses we identified SIRT1 as a target for inhibitory miR-132-3p control. These results are in accord with Hadar et al. (78), who reported a 4-fold lower expression of SIRT1 and a higher expression of miR-132 in Alzheimer’s disease patients. SIRT1 is a member of the sirtuins family, a group of mammalian class III histone deacetylases. Sirtuins were mainly investigated in the context of health span and longevity. SIRT1 controls mitochondrial, endocrine and hypothalamic functions (79–82). In addition, SIRT1 is involved in memory formation in the brain by promoting axonal elongation and dendritic branching and by modulating synaptic plasticity (83). Of particular relevance, SIRT1 has been widely investigated in the context of longevity and neuroprotection. Taken together, the identification of SIRT1 as a downstream target for miR-132-3p provides the physical foundation for the link between disruption of the GH-IGF1 axis and prolonged lifespan (84).

## Conclusions

The GH-IGF1 endocrine system has a critical role in determining lifespan, longevity and aging processes. We postulated that life-long deficiency of IGF1 in LS might activate cancer-protecting pathways at the organismal level, including apoptotic and autophagic mechanisms. In parallel, diminished IGF1 signaling might have a significant impact on nutrient sensing and response to oxidative stress, leading to an extended lifespan (*at least* in animal models). Our comprehensive analyses have identified a number of new targets for IGF1 action whose over- or under-representation in LS might be linked to cancer evasion and, possibly, extended lifespan. The worldwide dispersion of the small number of patients with genetic IGF1 deficiency hinders to reach a definite conclusion.

The identification of miR-132-3p as a top upregulated miR in LS is of major interest. We may envision a scenario in which low IGF1 concentrations in patients lead to enhanced miR-132-3p levels. In turn, this specific miR is directly responsible for SIRT1 inhibition and, most probably, additional gene expression. The transcriptional and epigenetic mechanisms that control the concerted expression of the IGF1-miR-132-3p-SIRT1 axis are yet to be dissected.

Finally, by mining genomic and epigenomic data from LS patients we might be able to generate new clinical information. This information will eventually translate into new avenues of research in the areas of aging, metabolism and oncology. We believe that our results may shed light on genetic and epigenetic events associated with increased lifespan in models of IGF1 deficiency. These studies might have a major translational impact in medicine.

## Author contributions

HW: Conceptualization, Data curation, Formal Analysis, Funding acquisition, Investigation, Project administration, Supervision, Writing – original draft, Writing – review and editing. ZL: Conceptualization, Investigation, Writing – review and editing.
